# Cusp-type bi-directional radiofrequency plasma thruster toward contactless active space debris removal

**DOI:** 10.1038/s41598-025-16449-9

**Published:** 2025-08-20

**Authors:** Kazunori Takahashi

**Affiliations:** Department of Electrical Engineering, Tohoku University, Sendai, 980-8579 Japan

**Keywords:** Plasma physics, Aerospace engineering

## Abstract

Active removal of space debris is an emerging technology aimed at sustaining space activity in Earth orbit by mitigating the risk of collisions between operational satellites and debris. Utilizing a bi-directional magnetic nozzle (MN) radiofrequency (rf) plasma thruster has been proposed to remediate and remove debris from the Earth orbit, where the debris is decelerated by continuously exerting a force to the debris by a plasma beam ejected from the thruster, and zero net force exerted to the thruster is simultaneously maintained by ejecting a second plasma beam to the opposite direction, maintaining the distance between the satellite and the debris. Previous laboratory experiment has demonstrated bi-directional plasma ejection from a single MN rf plasma thruster having two open source exits for symmetric magnetic field configuration having fairly straight magnetic field lines in the insulator source tube wound by a rf loop antenna. Enhancing the force exerted on the debris is essential for reducing the time required for deorbiting. Here it is demonstrated that a symmetric cusp-field configuration can increase the force exerted to the debris under the circumstance that the thrust is maintained at nearly zero level by the bi-directional ejection of the plasma, where the convergent-divergent MNs are formed near the two open-source exits.

## Introduction

Space debris are defined as human-made, non-functional objects, including fragments and parts of rockets and satellites launched over the last 50 years^1^. The number of objects around the low Earth Orbit (LEO) has increased drastically in recent years due to the frequent launches of satellites. Owing to their uncontrolled motion and its velocity exceeding that of bullets, space debris orbiting around Earth pose a serious threat by significant increase in the potential risk of collisions with satellites that support sustainable human activity in space. Investigations into the mass and size distributions have shown a continuously increasing quantity of debris in low Earth orbit (LEO)^2,3^. When the debris generation rate due to collisions exceeds the natural loss rate by orbital decay, the number of debris will continuously increase, being known as the Kessler syndrome^4^. The main concern in the context of Kessler Syndrome is larger debris than a meter. Accordingly, such large objects have properly been tracked and cataloged to prevent collisions with active satellites. Active debris removal (ADR) technologies targeting large debris have been investigated over the past few decades, employing robotic arms, nets, tether nets, and electrodynamic tether^5–7^. Moreover, a magnetic docking method is currently under development for deorbiting satellites at the end of their operational missions^8^. Since these methods are categorized as direct-contact approaches and carry the risk becoming entangled in the uncontrolled motion of debris, contactless methods have also been proposed and investigated, e.g., laser ablation, ion beams, and plasma beams^9–14^. In most contactless approaches, a deceleration force is applied to the debris either by irradiating it with an ion/plasma beam or by ablating surface material. Thereby the debris’ altitude can be lowered, eventually leading to re-enter into Earth’s atmosphere. It is obvious that the mission duration strongly depends on the magnitude of the deceleration force exerted to the debris.

**Fig. 1:**
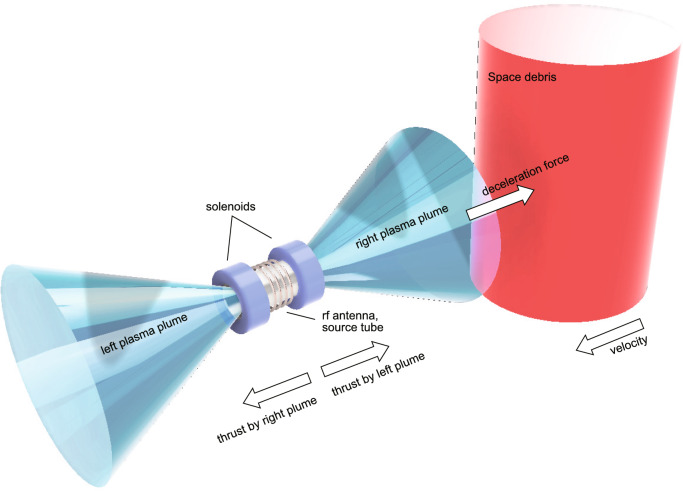
Concept of the ADR by using the MN rf plasma thruster, where the plasma plumes are ejected from both the right and left source exits. The deceleration force is exerted to the debris by irradiating the plasma plume ejected towards the debris, while zero net thrust exerted to the thruster is maintained by ejecting another beam to the opposite direction.

When a force is exerted on debris by using the ion/plasma beams ejected from an electric propulsion device, a reaction force (denoted as ‘thrust imparted by right plume’ in Fig. 1) acts on the satellite in the opposite direction (toward the left in Fig. 1), requiring the second beam ejection to the opposite direction (‘left plasma plume’ in Fig. 1) to compensate the reaction thrust as drawn in Fig. 1, by which the net thrust corresponding to sum of the thrusts induced by the right and left plasma plumes is maintained at zero level and the distance between the satellite and the debris can be maintained^10–13^. Previous studies have shown that a deceleration force of approximately 30 mN is typically required to deorbit debris of approximately a meter in size and a ton in mass within a period of 100 days^12^. A fundamental laboratory experiment using a radiofrequency (rf) plasma source under a magnetic nozzle configuration has shown bi-directional ion acceleration from the rf plasma source^15^. Subsequently, a single MN rf plasma thruster equipped with two open-source exits has successfully demonstrated the bi-directional plasma ejection^13^. In this experiment, a symmetric and fairly straight magnetic field lines have been applied to the source tube of the thruster, allowing a deceleration force to be exerted on a target simulating debris, while maintaining zero net thrust. Such a configuration has also been investigated using particle-in-cell simulations previously^16,17^. Furthermore, the fluxes of the plasma beams ejected to the two opposite directions can be controlled by adjusting the magnetic field configuration. This capability enables mode switching between acceleration, deceleration, and ADR simply by varying the solenoid currents of the single electric propulsion device.

Despite its potential for ADR applications of the MN rf plasma thruster, improving the thruster performance remains a significant challenge, as its performance is currently lower than those of more mature electric propulsion devices such as gridded ion thrusters and Hall thrusters^18,19^. In a previous laboratory experiment, the force exerted on the target plate located downstream of the thruster was limited to 8 mN, where the thruster had a 65-mm-diameter source tube and was operated at 1 kW rf power level^13^. In the MN rf plasma thruster, the high-density plasma created in the insulator source tube is guided along the magnetic field lines toward the source exit and expands along the divergent magnetic field, i.e., the MN. Numerous analytical, numerical, and experimental studies have revealed that the ions are spontaneously accelerated by an ambipolar electric field or a current-free double layer^20–24^. Furthermore, it has been identified that the axial momentum of the plasma is enhanced by the MN, where the Lorentz force arising from the azimuthal electron diamagnetic current and the radial magnetic fields converts the radial plasma momentum into the axial one^25–29^. The accelerated plasma beam is considered to detach from the MN via magnetohydrodynamic, unmagnetized, and wave-driven detachment processes as investigated vigorously^30–38^, while the detailed plasma detachment processes are still in argument. In the studies for performance improvement of the MN rf plasma thruster having a single source exit, the thrust has been enhanced by increasing the source tube diameter, by modifying the gas injector, and by applying a cusp magnetic field inside the source tube. As a result, the thruster efficiency is now approaching about 20–30$\%$^39,40^ for argon propellant. The performances of the gridded ion thrusters and the Hall thrusters are significantly lowered when alternating the limited xenon by the abundant argon, while a laboratory test has shown that the MN rf plasma thruster performed similarly with argon, krypton, and xenon^41^. This compatibility offers the potential for cost-effective ADR missions using readily available argon propellant and a single electric propulsion device.

In the present study, the cusp magnetic field is introduced inside the MN rf plasma thruster having two open-source exits. When the cusp magnetic field configuration is almost symmetric against the axial center where a rf antenna is installed, the convergent-divergent MN structures are formed for both source exits. Under this configuration, the plasma beams are ejected bi-directionally from the two open-source exits, enabling nearly zero net thrust while exerting a deceleration force on a target structure placed downstream of the thruster. It is observed that the force exerted on the target is larger with the cusp magnetic field than without it, thereby demonstrating the performance improvement of the MN rf plasma thruster in the ADR mode. The detected maximum force exerted on the target is about 25 mN at the rf power of 5 kW, approaching the force requirement in the ADR mission. Since the force is expected to depend on the distance between the debris and the satellites due to the plasma expansion along and detachment from the MN, further detailed investigation on the plasma expansion and detachment physics will be required as well as experimental validation in a large space simulation chamber. These remain important issues for future work.

## Experimental setup

**Fig. 2:**
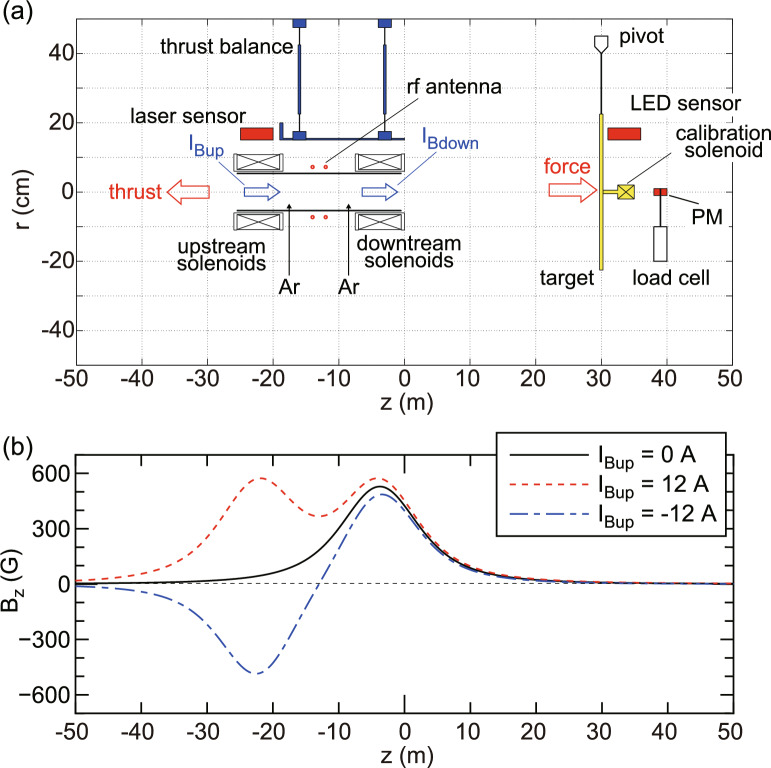
(**a**) Schematic diagram of the experimental setup installed inside the 1-m-diameter and 2-m-long cylindrical vacuum chamber. Directions of positive thrust and force to the debris, and magnetic field directions at the solenoid centers for $I_{Bup}>0$ and $I_{Bdown}>0$, are indicated by block arrows. (**b**) The calculated magnetic field strength on the *z* axis for ($I_{Bup}$, $I_{Bdown}$) = (0 A, 12 A) (a black solid line), (12 A, 12 A) (a red dotted line), and ($-12$ A, 12 A) (a blue dotted-dashed line). The thruster having two open-source exits is mounted on the pendulum thrust balance to measure the force exerted to the thruster, and the 45-cm-diameter pendulum target supported by a pivot is installed at $z=30$ cm to assess the deceleration force exerted to the target simulating debris.

**Fig. 3:**
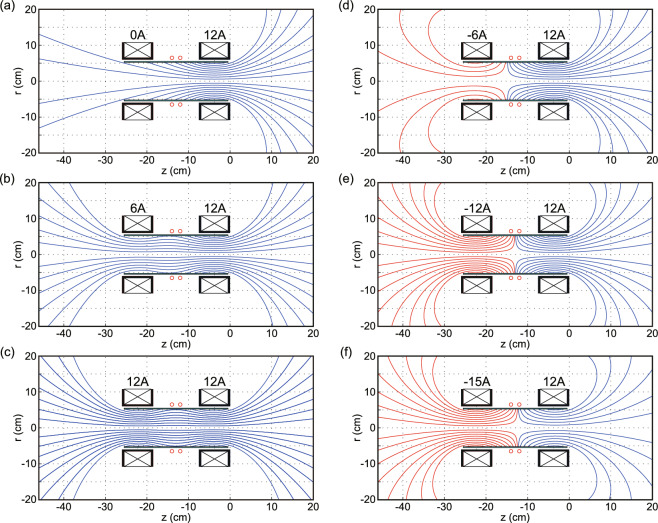
Calculated magnetic field lines for various combinations of ($I_{Bup}$, $I_{Bdown}$), where the rightward and leftward magnetic field lines are drawn by blue and red solid lines, respectively. The convergent-divergent magnetic nozzles are formed at the powered solenoid sides. The field lines are symmetric against the axial center of the source for ($I_{Bup}$, $I_{Bdown}$) = (12 A, 12 A) and ($-12$ A, 12 A), where the fairly straight field lines and the cusp are formed inside the source, respectively.

Experiments are performed in a 1-m-diameter and 2-m-long cylindrical vacuum chamber evacuated by three 3000 L/s turbomolecular pumping systems. Figure 2 shows the schematic diagram of the experimental setup, where the MN rf plasma thruster is installed inside the chamber. The thruster has a 11-cm-outer-diameter, 10.5-cm-inner-diameter, and 25-cm-long cylindrical glass source tube having two open exits. Two solenoids (638 turns for each), referred to as the upstream and downstream solenoids, are centered at $z=-22.25$ cm and $z=-3.75$ cm, respectively, where $z=0$ is defined as the right-hand side surface of the downstream solenoid holder, and the right-hand edge of the source tube is set at $z=-5$ mm. Argon gas is supplied through two gas injection holes on the side wall of the glass tube at $z=-8.5$ cm and $z=-17.5$ cm via a 1-mm-inner diameter and 2-mm-outer diameter ceramic tubes. The gas flow rates from each injection hole is maintained at 40 sccm (1.2 mg/s) using two independent mass flow controllers, giving the total gas flow rate of 80 sccm (2.4 mg/s). The argon pressure measured at the chamber side wall is about 30 mPa, and the effective pumping speed for argon is estimated to be 4500 L/s. A double-turn, water-cooled rf loop antenna wounds around the source tube at the axial center ($z=-13$ cm). The antenna is covered by an insulator and further shielded by a grounded metallic structure to suppress parasitic discharges around the antenna, as described in Ref.^42^. The dc solenoid currents ($I_{Bup}$, $I_{Bdown}$) are supplied to the upstream and downstream solenoids, respectively, by using independent dc power supplies. The calculated axial profiles of the magnetic field strength on the *z* axis for ($I_{Bup}$, $I_{Bdown}$) = (0, 12 A), (12 A, 12 A), and ($-12$ A, 12 A) are drawn by solid, dotted, and dotted-dashed lines, respectively, in Fig. 2b. Positive and negative solenoid currents produce rightward and leftward magnetic fields at their own centers, respectively, as drawn by block arrows in Fig. 2a. Figure 3 shows the calculated magnetic field lines for various combinations of ($I_{Bup}$, $I_{Bdown}$), illustrating the formation of convergent-divergent magnetic field lines near the powered solenoids. The rightward and leftward magnetic field lines are drawn by blue and red solid lines for clarity, respectively. It is found that the magnetic field configurations are axially symmetric with respect to the rf antenna position ($z=-13$ cm) for ($I_{Bup}$, $I_{Bdown}$) = (12 A, 12 A) and ($-12$ A, 12 A). In the former case, the magnetic field lines inside the source tube are relatively straight, while in the latter case, the cusp-shaped magnetic field lines are formed near the rf antenna.

After introducing the argon gas continuously and supplying the dc solenoid currents, the rf antenna is powered by a 13.56 MHz rf generator through an impedance matching box located outside the chamber and a water-cooled vacuum feedthrough, creating the plasma inside the source tube via inductive coupling. The impedance matching box has two variable high-voltage vacuum capacitors, and their capacitances are tuned so as to minimize the power reflection in advance. Detailed components for the thruster operation are further described in “Methods” section.

It is visually observed that the plasma plume ejected from the right source exit is significantly brighter than that from the left exit for the configuration of ($I_{Bup}$, $I_{Bdown}$) = (0, 12 A), indicating that the plasma is predominantly ejected from the side with the powered-solenoid side. In contrast, for the axially symmetric cases of ($I_{Bup}$, $I_{Bdown}$) = (12 A, 12 A) and ($-12$ A, 12 A), the plasma plumes from both sides appear visually similar, suggesting that the bi-directional plasma ejection is achieved under the axially symmetric magnetic field configurations, which contains either the straight magnetic fields or the cusp magnetic fields inside the source. Although photographs for these operations have not been taken, similar observations under no-cusp configuration have been reported previously^13^.

The plasma thruster described above is mounted on a pendulum thrust balance to evaluate the force exerted to the thruster, i.e., the thrust, as shown in Fig. 2a, where positive value of thrust is defined as the leftward direction (a block arrow in Fig. 2). The displacement of the thrust balance is measured using a high-resolution laser displacement sensor, providing the absolute value of the thrust by multiplying a calibration coefficient relating the displacement to the thrust. In addition, a 45-cm-diameter target plate made of compressed mica is suspended from a pivot located at $z=30$ cm. The axial force exerted on the target plate can be obtained by multiplying a calibration coefficient to the target displacement induced by the plasma production, which is measured by using a light-emitting-diode (LED) sensor. Positive value of force to the target is defined as the rightward direction as indicated by a block arrow in Fig. 2. The detailed procedures for the calibrations of the thrust balance and the target balance can be found in the “Methods” section.

For the assessment of the thrust and the force to the target, argon gas is continuously introduced from the two gas injection ports in advance. After the pressure measured at the chamber side wall is in a steady state, the solenoid currents ($I_{Bup}$, $I_{Bdown}$) are turned on and the signals from both the laser and LED sensors are taken for 5–10 s to identify the equilibrium positions of the thrust balance and the target. Subsequently, the rf power $P_{rf}$ and the plasma is turned on, and the signals from the sensors are further taken for 5–10 s. Finally, the rf power and the solenoid currents are turned off in order. The displacements of the thrust balance and the target, which are induced by only the plasma, are estimated from the differences between the equilibrium positions before and after turning on the rf power. This procedure allows to assess instantaneous values of the thrust and the force to the target, which are induced by only the plasma. Therefore, the measurements do not include influence of the magnetic fields, and the forces by the cold gas flow. The thrust and the force to the target induced by the cold gas flow can be briefly estimated from the mass flow rate ${\dot{m}}$ and their velocity $v_g$ as ${\dot{m}}v_g$. Assuming the mass flow rate of 1.2 mg/s and the velocity of 300 m/s, the forces by the cold gas flow is estimated as less than 0.5 mN, which is much smaller than the detected thrust and force to the target in the present experiment.

**Fig. 4:**
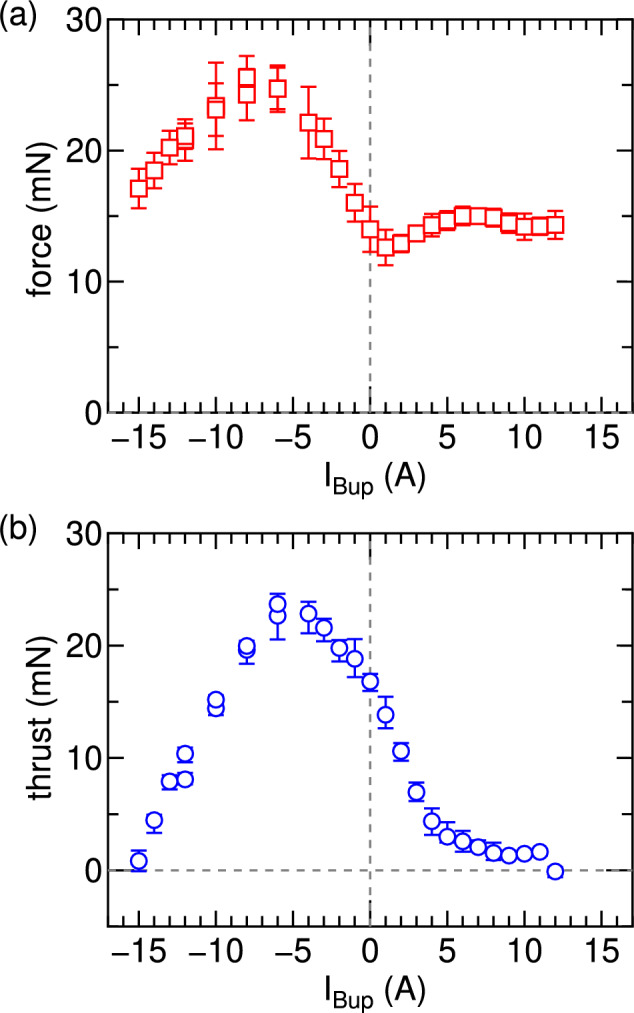
(**a**) Axial force exerted on the target plate and (**b**) thrust exerted on the thruster as functions of the upstream solenoid current $I_{Bup}$ for the downstream solenoid current of $I_{Bdown}=12$ A and the rf power of $P_{rf}=3$ kW. The error bars of the solenoid current is $\pm 5~\%$ of the output current, which is less than the size of the marks in the figures. The maximum thrust is obtained for $I_{Bup}\sim -6$ A, which is consistent with the previous experiment for the single source exit^40^, providing the thruster acceleration mode. The leftward thrusts are detected for all the conditions, while nearly zero thrust is observed for $I_{Bup}=-15$ A and $I_{Bup}=12$ A, providing the debris removal mode. The force exerted on the target for $I_{Bup}=-15$ A is larger than that for $I_{Bup}=12$ A, indicating that the better performance for the ADR mode can be obtained by applying the cusp inside the source.

## Experimental results

Figure 4 shows (a) the measured force exerted on the target plate and (b) the thrust exerted on the thruster as functions of the upstream solenoid current $I_{Bup}$, for the fixed downstream solenoid current of $I_{Bdown}=12$ A and the rf power of $P_{rf}=3$ kW, where the direction of the positive values are defined as the rightward and leftward directions for the force to the target (Fig. 4a) and thrust (Fig. 4b), respectively. It is found that both forces to the target and the thruster have their maxima for the condition of $I_{Bup}\sim -6$ A, where the cusp magnetic field is formed on the left-hand side of the rf antenna as drawn in Fig. 3d. In the previous experiment for the thrust having the single open source exit, the maximum thrust has been obtained by locating the cusp magnetic field between the rf antenna and the source back wall, which is considered to be due to the geometrical isolation of the plasma from the wall at approximately left half of the source tube and the resultant reduction of the plasma loss to the wall^40^. The present result is consistent with the previous observation and interpretation. As previously described, the distance between the debris and the spacecraft cannot be maintained for this operation mode, as the leftward thrust is generated by ejecting the plasma plume to the right-hand side. This condition of ($I_{Bup}$, $I_{Bdown}$) $\sim$ ($-6$ A, 12 A) is appropriate for the acceleration mode propelling a spacecraft to the opposite direction to the debris. Conversely, the deceleration mode propelling the spacecraft toward the debris can be achieved by interchanging the values of $I_{Bup}$ and $I_{Bdown}$ as previously demonstrated^13^.

As in the previous experiment on the bi-directional plasma ejection, the thrust significantly decreases with an increase in $I_{Bup}$ for $I_{Bup}>0$, approaching nearly zero at $I_{Bup}\sim 12$ A, while a fairly constant force to the target is maintained. Most of the field lines passing near the wall at the antenna position are terminated by the upstream radial wall for $I_{Bup}=0$ A, resulting in the major plume ejection from the right-hand source exit due to the left-hand plasma termination at the radial wall. For $I_{Bup}>0$, some of the field lines can intersect the left-hand open-source exit as drawn in Fig. 3b, yielding the leftward plume ejection and the reduction of the net thrust. Since the field lines intersecting the wall at the antenna position are still close to the radial wall at the left half of the source tube, confinement effect of the magnetic fields in the left half of the source is not significant, showing the slight increase in the force for $I_{Bup}\sim 0$-5 A and the fairly constant force to the target for $I_{Bup}>5$ A (Fig. 4a). The condition of zero net thrust corresponds to the symmetric magnetic field configuration against the source tube center with the fairly straight magnetic field lines inside the source. Under this condition, the measured force exerted to the target is $14.3\pm 1$ mN, as shown in Fig. 4a. These results indicate that the deceleration force can be exerted on the debris under the circumstance that zero net thrust to the spacecraft is sustained, which is suitable for the ADR mode.

Interestingly, the thrust also decreases with increasing the magnitude $|I_{Bup}|$ of the negative upstream solenoid current, i.e., for $I_{Bup}<-7$ A, and nearly zero thrust can be obtained for $I_{Bup}=-15$ A, where the cusp field is formed near the rf antenna position as seen in Fig. 3f. This operation mode is also used for the ADR mode, like the ($I_{Bup}$, $I_{Bdown}$) $=$ (12 A, 12 A) case. The force exerted on the target for ($I_{Bup}$, $I_{Bdown}$) $=$ (− 15 A, 12 A) is $17.1 \pm 1.5$ mN, being about 20 $\%$ greater than that obtained for the (12 A, 12 A) case. The results demonstrate that the presence of the cusp field near the rf antenna enhances the deceleration force exerted on the debris compared to the case with the symmetric straight magnetic field lines in the source. It is worth noting that the upstream current magnitude required to achieve zero thrust ($I_{Bup}=-15$ A) is slightly different from the downstream current ($I_{Bdown}=12$ A). It is noted that a similar result has been obtained when changing the field direction, i.e., for ($I_{Bup}$, $I_{Bdown}$) $=$ (− 15 A, 12 A). This discrepancy is likely due to a misalignment between the rf antenna and the solenoids, suggesting that the cusp-field-based ADR mode is more sensitive to structural alignment than the straight-field configuration. The topologies of the magnetic field lines in Fig. 3f still show the existence of the cusp in the antenna region. More precise design and fabrication of the thruster structure or adjustment of the solenoid currents is necessary to maintain precisely zero net thrust.

**Fig. 5:**
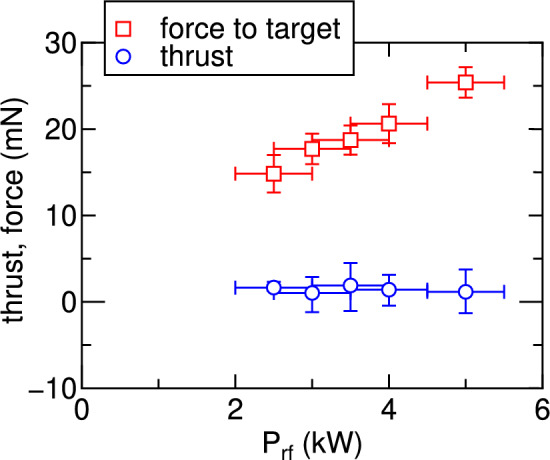
Measured force to the target (open squares) and thrust (open circles) as functions of the rf power $P_{rf}$ for ($I_{Bup}$, $I_{Bdown}$) $=$ (-15 A, 12 A). The force to the target increases with an increase in $P_{rf}$, while nearly zero thrust is maintained. The maximum deceleration force of about 25 mN is obtained for 5 kW rf power, approaching the force requirement in the debris removal mission.

Here, the enhancement of the deceleration force with the cusp field inside the source is discussed in relation to the magnetic field topologies shown in Fig. 3c,e. The plasma generated by the antenna fields is transported along the magnetic field lines. A comparison of the field topologies in Fig. 3c,e reveals that, although the field lines intersecting the radial wall near the rf antenna converge around the solenoid centers in both cases, the field lines in Fig. 3e traverse a more central region of the source tube than those in Fig. 3c. This suggests that the plasma is more effectively isolated from the radial source wall near the axial locations of the solenoid, thereby reducing the plasma and energy losses to the wall. As a result of the reductions of the plasma and energy losses, the momentum transferred to the target would be enhanced when the cusp field is formed near the rf antenna.

For the ADR mode with the cusp field near the antenna, corresponding to ($I_{Bup}$, $I_{Bdown}$) $=$ ($-15$ A, 12 A), both the thrust and the force exerted on the target are measured as functions of the nominal rf power $P_{rf}$, as plotted by open circles and squares in Fig. 5, respectively. The variable capacitors in the matching box are tuned in each case so as to eliminate detectable power reflection, i.e., the horizontal axis corresponds to the rf power delivered to the load including the matching box, the antenna, and the plasma. The results show that the force exerted on the target can be increased to $\sim 25$ mN while maintaining the net thrust at a near-zero level. It should be noted that the thrust is not exactly zero, likely due to the misalignment in the thruster settings (e.g., solenoids and antenna positioning). Although more precise mechanical alignment and design optimization will be necessary to achieve strictly zero thrust, the present experiment demonstrate that the cusp-field configuration enables the deceleration force approaching the requirements for the ADR mission.

The ADR mission requires two electric propulsion devices for the thrust cancellation, while the MN rf plasma thruster can achieve it by the single thruster. Therefore, the specific impulse $I_{sp}$ and the thruster efficiency $\eta _T$ are calculated from the half power and the half mass flow rate, where typical electric power for the single solenoid is about $P_{sol}=320$ W for the 12 A current. Using the force of $F = 25$ mN, the argon mass flow rate of ${\dot{m}}=1.2$ mg/s for one gas injection port, and the half rf power of $P_{rf}=2.5$ kW,

1 $$\begin{aligned} I_{sp}= & \frac{F}{{\dot{m}} g} \sim 2100~\text {s}, \end{aligned}$$

2 $$\begin{aligned} \eta _{T}= & \frac{F^2}{2 {\dot{m}} (P_{rf}+P_{sol})} \sim 10~\%, \end{aligned}$$

where *g* is the gravitational acceleration. It is well known that the specific impulse and the thruster efficiency of the Hall thruster are degraded for argon, e.g., $I_{sp} \sim 1000-1500$ s and $\eta _{T} \sim 10\%$ have been reported^43^, being similar to the performance estimated here. Therefore, the bi-directional MN rf plasma thruster could be one of the candidates for electric-propulsion-based ADR method.

It should be mentioned that the target simulating orbital debris is located 30-cm downstream from the thruster. As the plasma expands along the MN, the plume diameter increases with axial distance. When the debris is located farther from the thruster exit, it is expected that the plume diameter may exceed the size of the debris. In such cases, only a portion of the plasma plume impinges on the debris surface, leading to a significant dependence of the exerted force on both the distance between the thruster and the debris and the debris size. This behavior will also be influenced by the plasma detachment process from the MN and the resultant plume divergence. As observed in the previous experiments, the ions deviate from the MN at several tens of centimeters downstream of the MN, and exhibit a divergence angle smaller than that of the magnetic field lines^37,44^. One of the experiments suggests that the deviating ions are neutralized by the electrons transported across the magnetic field lines due to the wave-driven cross-field transport. In earlier work, collimated ion beams accelerated via the current-free double layer have also been observed^45^. These results indicate that the divergence of the plasma plume is closely related to the acceleration and detachment processes occurring in the MN. However, the physics of the plasma detachment, in particular, remains incompletely understood. To accurately estimate the deceleration force exerted on debris located at greater distances, comprehensive study involving large-scale space simulation chamber experiments and numerical analysis will be necessary.

In the present experiment, the target plate is made of the insulator, which would become charged to a floating potential by irradiating the plasma plume. The surface charge might influence the force measurements due to a coulomb force. However, the previous experiment has reported that the thrust measured by the thrust balance shows a good agreement with that obtained by the insulator target technique^46^. This fact demonstrates that the momentum delivered to the target is equivalent to that ejected from the thruster, indicating a negligible influence of the surface charge on the force integrated over the surface area.

## Conclusion

The symmetric cusp magnetic field is applied near the rf antenna of the MN rf plasma thruster having two open-source exits, forming convergent-divergent magnetic fields around the two solenoids located near the thruster exits. Under this configuration, the MNs are established on both the upstream and downstream sides of the thruster. Exerting the deceleration force on the target and maintaining near-zero net thrust are simultaneously achieved for both the magnetic field configurations having the cusp and no cusp, where the plasmas are bi-directionally ejected from the two open-source exits. This feature allows for the contactless removal of space debris. The deceleration force exerted on the target simulating space debris is enhanced by about 20$\%$ in the symmetric cusp field configuration in comparison to the symmetric no-cusp field configuration. This can be qualitatively interpreted with the reduced plasma loss to the wall, owing to improved magnetic confinement and geometrical isolation of the plasma from the wall by more convergent field lines in the cusp configuration. The maximum force exerted to the target is increased up to about 25 mN for the rf power of 5 kW, while maintaining the thrust level close to zero.

## Methods

### Thruster components

**Fig. 6:**
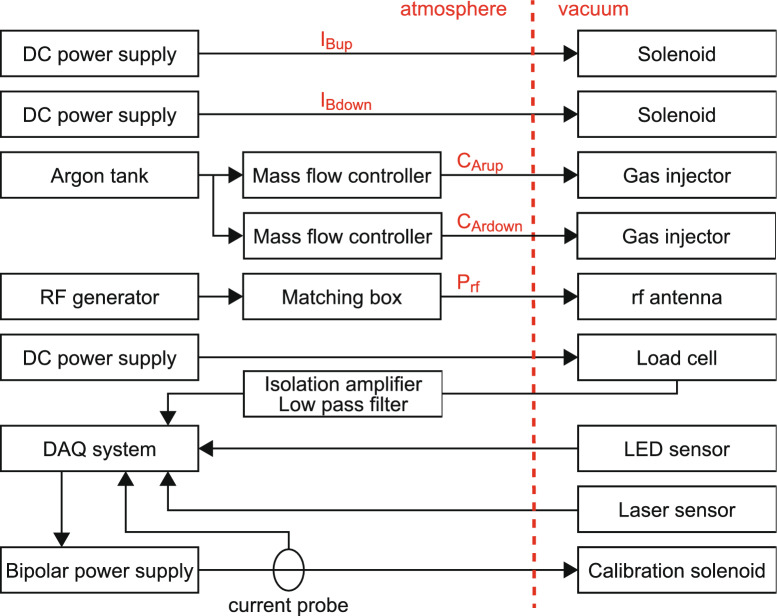
Detailed components for the thrusters, the power supplies, the target balance, and the thrust balance.

**Fig. 7:**
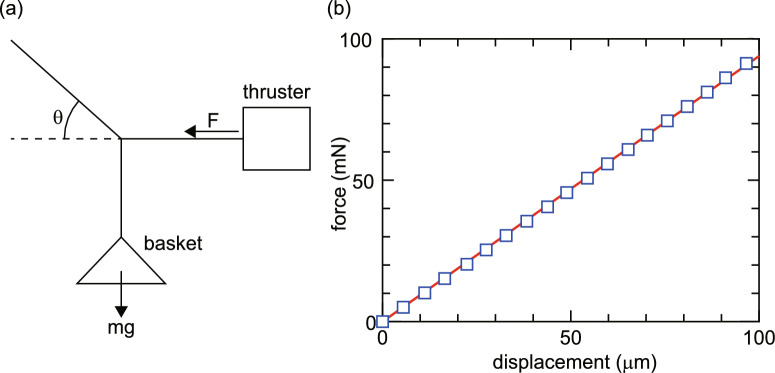
(**a**) Mechanics for the thrust balance calibration. Known mass pieces are put on the basket and the displacements are measured by the laser displacement sensor. (**b**) Measured displacement versus force calculated by the simple mechanics (open squares). The measured data are well fitted by a linear solid line giving a calibration coefficient of $\sim 0.94~\mathrm{mN/\mu m}$.

Figure 6 shows the components of the thruster, including the power supplies, thrust balance, and target balance. The components shown on the left-hand and right-hand sides of the diagram are located outside and inside the chamber, respectively. The upstream and downstream solenoids are independently powered by the dc power supplies through vacuum feedthroughs. Argon gas from a storage tank is delivered to the two mass flow controllers, which are connected to the upstream and downstream gas injectors installed on the side wall of the thruster glass tube. The gas is continuously introduced from the two gas injectors with the constant gas flow rates of 40 sccm, resulting in the total gas flow rate of 80 sccm. The rf power is supplied from the 13.56 MHz rf generator to the rf antenna via the impedance matching box having two variable capacitors and a fixed capacitor. The housing of the matching box is directly connected to the chamber side port and the output terminals are connected to water-cooled feedthroughs, which are further connected to the rf loop antenna made of copper tube; enabling water cooling to maintain the antenna temperature at approximately room temperature ($\sim 300$ K). The feedthrough and the exterior of the rf antenna are shielded by a grounded metallic structure to suppress parasitic discharges between the antenna and the grounded vacuum chamber, where the antenna is mechanically supported by insulator parts connected to the grounded shield structure.

A data acquisition (DAQ) board (NI-USB-6343), controlled by Labview programs, is used to acquire the signals from the laser displacement sensor (for the thrust balance) and the LED displacement sensor (for the target balance). For calibration of the target balance, an electromagnetic force is applied between the target and the permanent magnet (PM) mounted on the load cell. This force is regulated by the electric current supplied to the calibration solenoid installed on the rear side of the target, where a bipolar power supply is employed to precisely control the calibration current. It is noted that the load cell is powered by a low noise regulated dc voltage power supply to minimize noise signals. The signal output from the load cell is amplified by using a precision isolation amplifier with a gain of 1000 and passed through a 10 Hz low pass filter. Both the calibration current and the amplified signal corresponding to the force exerted to the load cell are simultaneously measured by the DAQ system.

**Fig. 8:**
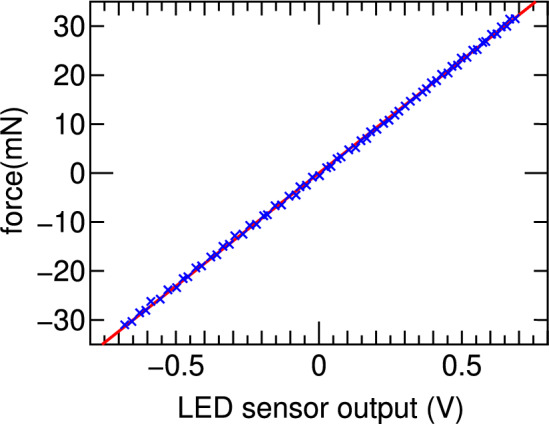
Measured displacement of the target plate versus force exerted to the PM mounted on the load cell (blue crosses). The data can be fitted by a linear line (a red solid line) giving the calibration coefficient relating the LED sensor output to the force exerted to the target.

### Thrust balance calibration

The thrust balance consists of a double pendulum system as drawn in Fig. 2a. To calibrate the thrust balance, a calibration basket is suspended by a horizontal thread attached to the left-hand side of the thruster, and a second thread fixed to the support structure as drawn in Fig. 7a. Known mass pieces are placed on the calibration basket, and then the resultant displacement of the thrust stand is measured by a laser displacement sensor. The horizontal force *F* applied by the mass *m* can be calculated from simple mechanics as $F = mg \cot {\theta }$, where $\theta$ and *g* are the angle between the horizontal and second threads, and the gravitational acceleration, respectively. The measured displacement as a function of the applied horizontal force is plotted by open squares in Fig. 7b and is well-fitted by a linear line as drawn by a solid line. The relationship yields a calibration coefficient of $\sim 0.94~\mathrm{mN/\mu m}$, which relates the measured displacement to the absolute value of the horizontal force, i.e., the thrust.

### Target balance calibration

A small solenoid is mounted on the backside of the pendulum target plate, and the small PM is affixed to the load cell located on the mechanical support structure for the purpose of calibrating the target balance, where the displacement of the target plate is measured by using the commercial light-emitting-diode (LED) displacement sensor. When the calibration solenoid is powered by a bipolar power supply, it generates the axial magnetic fields that exerts a force on the PM. Since the reaction force is exerted on the small solenoid mounted on the target, the magnitude of the force exerted to the target structure is equivalent to that exerted to the PM, which can be measured by the load cell. The calibration solenoid current is swept from $+1.5$ A to $-1.5$ A very slowly (for about 20 s); the signal from the load cell and the displacement signal from the LED sensor are simultaneously digitized by the DAQ system. The measured displacement is plotted as blue crosses in Fig. 8 as a function of the applied axial force. The relationship can be well-fitted by a linear line giving a calibration coefficient of $\sim 46.1~\mathrm{mN/V}$.

## Data Availability

The data that support the figures within this paper are available from corresponding author upon reasonable request.
